# iUMRG: multi-layered network-guided propagation modeling for the inference of susceptibility genes and potential drugs against uveal melanoma

**DOI:** 10.1038/s41540-022-00227-8

**Published:** 2022-05-24

**Authors:** Yueping Ren, Congcong Yan, Lili Wu, Jingting Zhao, Mingwei Chen, Meng Zhou, Xiaoyan Wang, Tonghua Liu, Quanyong Yi, Jie Sun

**Affiliations:** 1 School of Biomedical Engineering, School of Ophthalmology & Optometry and Eye Hospital, Wenzhou Medical University, Wenzhou, 325027 P. R. China; 2 Tibet Medical College, Beijing University of Chinese Medicine, Tibet, 850010 P. R. China; 3 Department of Human Anatomy, Harbin Medical University, Harbin, 150081 P. R. China; 4 The Affiliated Ningbo Eye Hospital of Wenzhou Medical University, Ningbo, 315042 P. R. China

**Keywords:** Computational biology and bioinformatics, Biochemical networks

## Abstract

Uveal melanoma (UM) is the most common primary malignant intraocular tumor. The use of precision medicine for UM to enable personalized diagnosis, prognosis, and treatment require the development of computer-aided strategies and predictive tools that can identify novel high-confidence susceptibility genes (HSGs) and potential therapeutic drugs. In the present study, a computational framework via propagation modeling on integrated multi-layered molecular networks (abbreviated as iUMRG) was proposed for the systematic inference of HSGs in UM. Under the leave-one-out cross-validation experiments, the iUMRG achieved superior predictive performance and yielded a higher area under the receiver operating characteristic curve value (0.8825) for experimentally verified SGs. In addition, using the experimentally verified SGs as seeds, genome-wide screening was performed to detect candidate HSGs using the iUMRG. Multi-perspective validation analysis indicated that most of the top 50 candidate HSGs were indeed markedly associated with UM carcinogenesis, progression, and outcome. Finally, drug repositioning experiments performed on the HSGs revealed 17 potential targets and 10 potential drugs, of which six have been approved for UM treatment. In conclusion, the proposed iUMRG is an effective supplementary tool in UM precision medicine, which may assist the development of new medical therapies and discover new SGs.

## Introduction

Uveal melanoma (UM) is the most common primary malignant intraocular tumor in adults, affecting ~5/1,000,000 individuals^1^. Current treatments for the local primary tumor include enucleation and radiation therapy (RT)^2,3^. However, the indication depends on the tumor size and location relative to the adjacent ocular tissues, as well as the existence of comorbidities. The incidence of distant metastases is as high as 50% within 10 years of initial diagnosis, and the prognosis of metastatic disease is poor, with a reported median overall survival (OS) of only 6-12 months^4^. The distant metastasis and mortality rates have remained unchanged in the last decades, although surgical techniques and RT have improved.

There are currently no definitive treatments available for metastatic UM. Several clinical trials have demonstrated that conventional cytotoxic chemotherapy was ineffective for metastatic UM^5,6^. Even the emerging targeted therapies, which target associated genetic mutations and downstream signaling pathways, have not yet yielded determinate results^7,8^. It is therefore critical to perform genetic mapping and subsequently to identify new candidate cancer biomarkers and treatments. However, the current knowledge for susceptibility genes identification is mainly derived from preclinical in vitro or in vivo studies, which are limited, since these experimental models cannot fully recapitulate the clinical situation^9^. With the advent of network medicine, the large amount of available biomedical data provides a chance to build a framework that integrates preclinical results through highly efficient networks^10^.

In the present study, a heterogeneous multi-layered molecular network (HMMN) was constructed by integrating multiple data resources (transcriptomics, ncRNAomics, regulatomics, and interactomics). Next, a network propagation algorithm was used for the systematic inference of novel susceptibility genes (SGs) in and potential drugs for UM. The relevance between the candidate SGs and UM carcinogenesis, progression, and outcome was evaluated. Finally, candidate targets and drugs of these novel SGs were inferred using the drug repositioning approach.

## Results

### Reconstruction of the HMMN

To re-construct a comprehensive molecular network encompassing as many potential regulatory edges as possible, different molecular networks were first assembled using publicly available transcriptomics, ncRNAomics, regulatomics, and interactomics data (Supplementary Table 1). A lncRNA-mRNA network with 1954 nodes and 2261 edges was constructed from lncRNAtarget and starBase. miRNA-lncRNA and miRNA-gene regulatory networks were constructed from lncBase and miRTarBase, including 6701 nodes and 48,182 edges, and 3664 nodes and 8641 nodes, respectively. Two TF-miRNA and TF-gene regulatory networks were constructed from TransmiR and TRANSFAC, including 784 nodes and 3578 edges, and 2322 nodes, and 6246 edges, respectively. A protein–protein interaction network was constructed from HuRI, including 9060 nodes and 63,242 edges. Finally, an HMMN with 18,231 nodes and 12,6187 edges was re-constructed by integrating these heterogeneous networks.

### Prediction of novel SGs using the iUMRG

**Fig. 1:**
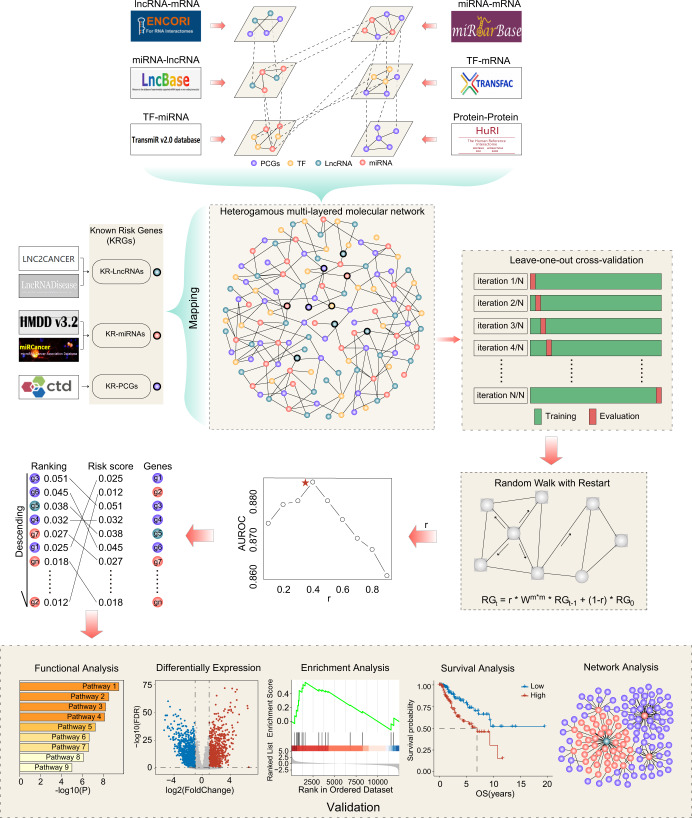
Illustration of the overall framework of iUMRG.

**Fig. 2:**
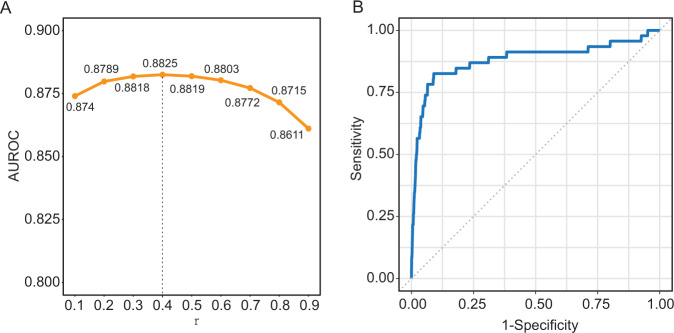
Performance evaluation of the iUMRG. **A** AUC values of r under each LOOCV. **B** Receiver operating characteristic curves of iUMRG under LOOCV based on 59 experimentally supported UM-related susceptibility genes. AUC receiver operating characteristic curve, UM uveal melanoma, LOOCV leave-one-out cross-validation.

The workflow of a computational framework (hereinafter referred to as iUMRG) via propagation modeling for detecting SGs in UM is illustrated in Fig. 1. As shown in Fig. 1, 59 experimentally supported UM-related SGs were first collected, including 8 mRNAs, 39 miRNAs, and 12 lncRNAs. Next, random walk with restart-based network propagation algorithm was used in the HMMN and LOOCV to train iUMRG with different *r* values by considering one known UM-related gene as a seed node and 58 other known UM-related genes as testing cases. ROC analysis demonstrated that the iUMRG achieved the highest predictive performance with a ROC curve (AUC) = 0.8825 at an *r* = 0.4 (Fig. 2A, B).

All 59 known SGs were used as seed nodes to predict novel UM-related SGs, and iUMRG was used on the HMMN. Finally, all nodes in the HMMN were ranked according to the risk score, and the top 50 genes were defined as the most likely novel SGs, including 22 protein-coding genes, 18 miRNAs, and 10 lncRNAs (Supplementary Table 3).

### Functional analyses of novel UM-related SGs

**Fig. 3:**
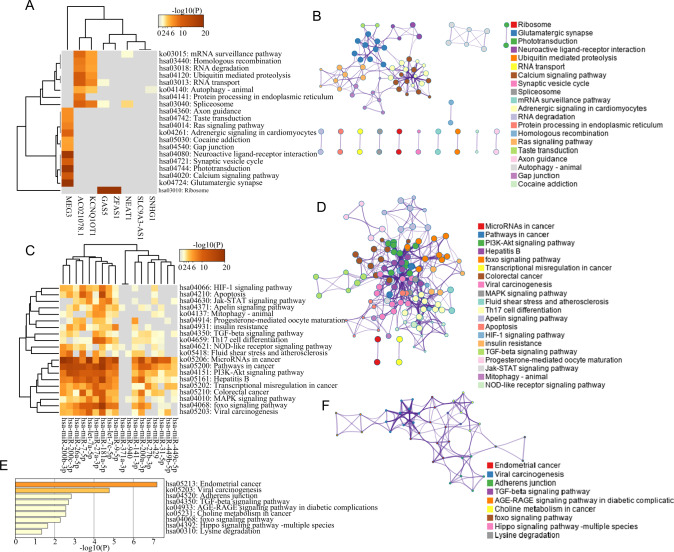
Functional analysis of novel UM-related susceptibility genes. Clustered heatmap of top 20 enriched pathways for novel UM-related risk **A** lncRNAs and **C** miRNAs, colored by P-values. Network of enriched pathways are colored by functional classes and nodes that share the same cluster are typically close to each other for novel UM-related risk **B** lncRNAs, **D** miRNAs and **F** mRNAs. **E** Metascape bar graph for viewing enriched pathways of mRNAs, colored by *P*-values. UM uveal melanoma, lncRNA long non-coding RNA, miRNAs microRNA, AGE-RAGE advanced glycation end products-receptor of RAGE.

KEGG enrichment analysis was performed for the co-expressed genes of 10 novel risk lncRNAs, and it was found that they were significantly enriched in several functional clusters, including calcium signaling pathway, homologous recombination, neuroactive ligand-receptor interaction, and autophagy (Fig. 3A, B). Next, KEGG enrichment analysis was performed for the target genes of 18 novel UM-related risk miRNAs, and it was demonstrated that viral carcinogenesis, miRNAs in cancer, pathways in cancer and transcriptional misregulation in cancer were significantly enriched (Fig. 3C, D). KEGG enrichment analysis was then performed for novel risk protein-coding genes, and it was found that these predicted novel risk protein-coding genes were involved in viral carcinogenesis (Fig. 3E, F). These functional clusters enriched by novel UM-related SGs were associated with known biological pathways involved in cancer carcinogenesis and progression, which supported the potential functional roles of these novel SGs in UM.

### Further confirmation of novel UM-related SGs in different patient cohorts

**Fig. 4:**
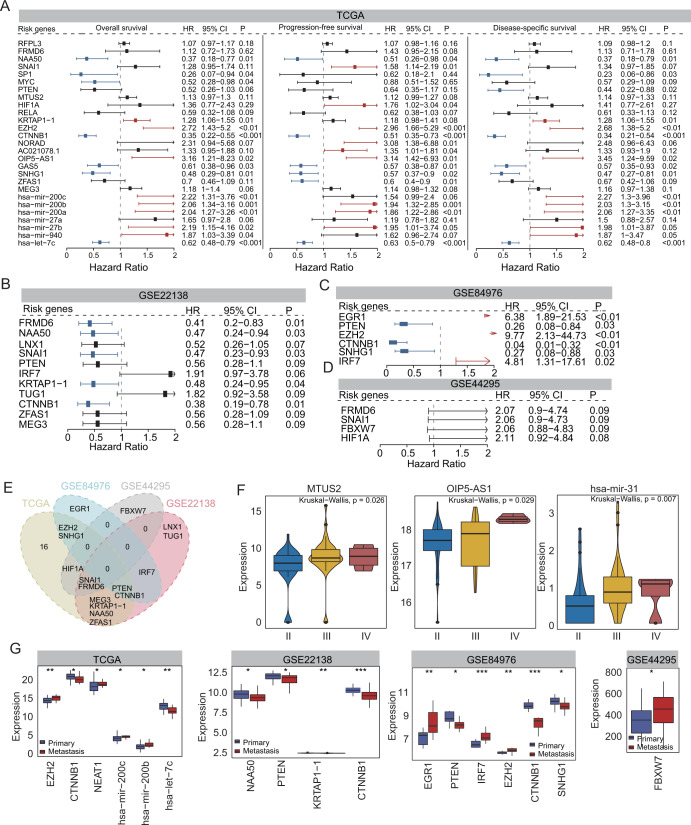
Clinical validation of novel UM-related SGs. Forest plot visualizing the hazard ratios (95% confidence interval) of univariate Cox regression analysis for novel UM-related SGs in **A** TCGA, **B** GSE22138, **C** GSE84976, and **D** GSE44295 cohorts. **E** Venn diagram illustrating overlaps of prognostic genes among different patient cohorts. **F** Boxplot showing expression levels of stage-related SGs in TCGA cohort. **G** Boxplot showing expression levels of metastasis-related SGs in different patient cohorts. UM uveal melanoma, SGs susceptibility genes, TCGA The Cancer Genome Atlas.

The association of these novel SGs with UM progression and metastasis was further examined in several published patient cohorts. First, univariate Cox regression analysis was performed to examine the association of these 50 novel SGs with OS, progression-free survival (PFS), and disease-specific survival in the TCGA cohort, and it was found that 27/50 candidate SGs were significantly and marginally significantly associated with patient outcome (Fig. 4A). A similar analysis was also performed for the other three patient cohorts. A total of 11, 6, and 4 candidate SGs were found to be significantly and marginally significantly associated with patient outcome (Fig. 4B–E). Further analysis in the TCGA cohort revealed that expression levels of three candidate SGs (*MTUS2*, *OIP5-AS1*, and *has-mir-31*) are significantly different among different clinical stages of UM patients (*P* = 0.026 for *MTUS2*, *P* = 0.029 for *OIP5-AS1* and *P* = 0.007 for *has-mir-31*) (Fig. 4F). Specifically, the increased expression levels of these three candidate SGs are associated with UM progression (Fig. 4F). Further comparative analysis of expression patterns between primary and metastatic tumors showed that 13 candidate SGs were significantly differentially expressed between primary and metastatic tumors in at least one of the four cohorts (TCGA, GSE22138, GSE84976, and GSE44295) (Fig. 4G). Specifically, seven candidate SGs (*EZH2*, *NEAT1*, *hsa-mir-200c*, *hsa-mir-200b*, *EGR1*, *IRF7,* and *FBXW7*) are significantly up-regulated and six (*CTNNB1*, *KRTAP1-1*, *NAA50*, *PTEN*, *SNHG1,* and *hsa-let-7c*) are significantly down-regulated in metastatic UM tumors compared with primary tumors (Fig. 4G). Based on the above observation, 35/50 candidate SGs were shown to be associated with UM progression, metastasis, and clinical outcome.

### Network analyses of novel UM-related SGs

**Fig. 5:**
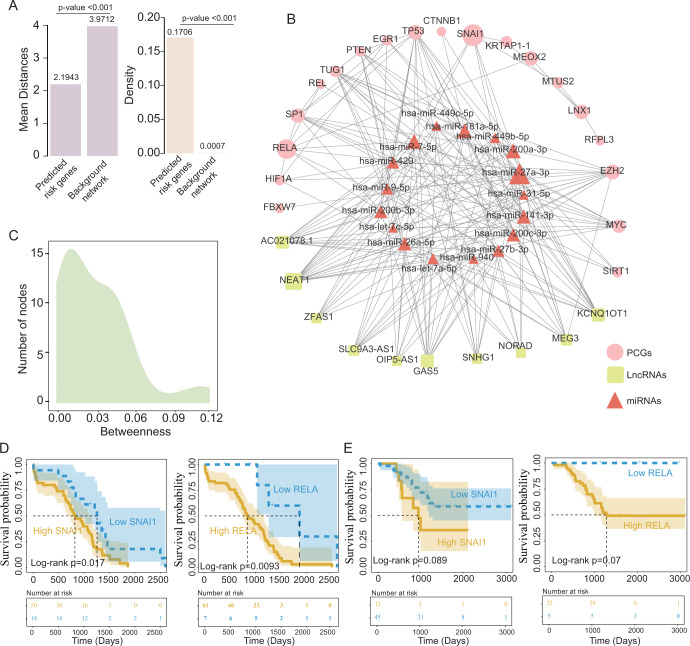
Network analysis of novel UM-related SGs. **A** Histogram showing the distribution of mean distances and density of predicted SGs and background network. **B** Subnetwork of novel UM-related SGs. **C** Distribution of betweenness of nodes in the subnetwork. UM uveal melanoma, SGs susceptibility genes. lncRNA long non-coding RNA, miRNA/miR microRNA, PCGs protein-coding genes.

Network features were further examined using the HMMN as a background network; these 50 novel SGs tended to have smaller mean distances and higher densities than other gene nodes in the background network (Fig. 5A). Furthermore, these 50 novel SGs could form a sub-network with 46 nodes and 201 edges (Fig. 5B). Of the 46 nodes, snail family transcriptional repressor 1 (*SNAI1*) and RELA proto-oncogene, NF-κB subunit (*RELA*) were found to have the highest betweenness centrality, suggesting that these two genes were more likely to be important nodes in this subnetwork (Fig. 5C). When examining the association of *SNAI1* and *RELA* with patient prognosis in the TCGA cohort, it was found that the expression levels of *SNAI1* and *RELA* were capable of distinguishing between patients with substantially different outcomes. As shown in Fig. 5D, patients with a high *SNAI1*/*RELA* expression tended to be at an increased risk of a poor outcome compared with those with a low expression (*P* = 0.017 for *SNAI1* and *P* = 0.0093 for *RELA*; log-rank test). A similar prognostic role of *SNAI1* and *RELA* was also observed in the GSE44295 dataset. Patients with a low *SNAI1*/*RELA* expression had marginally significantly improved survival compared with those with a high *SNAI1*/*RELA* expression (*P* = 0.089 for *SNAI1* and *P* = 0.07 for *RELA*; log-rank test; Fig. 5E).

### Prediction of targets and corresponding drugs for novel UM-related SGs

**Fig. 6:**
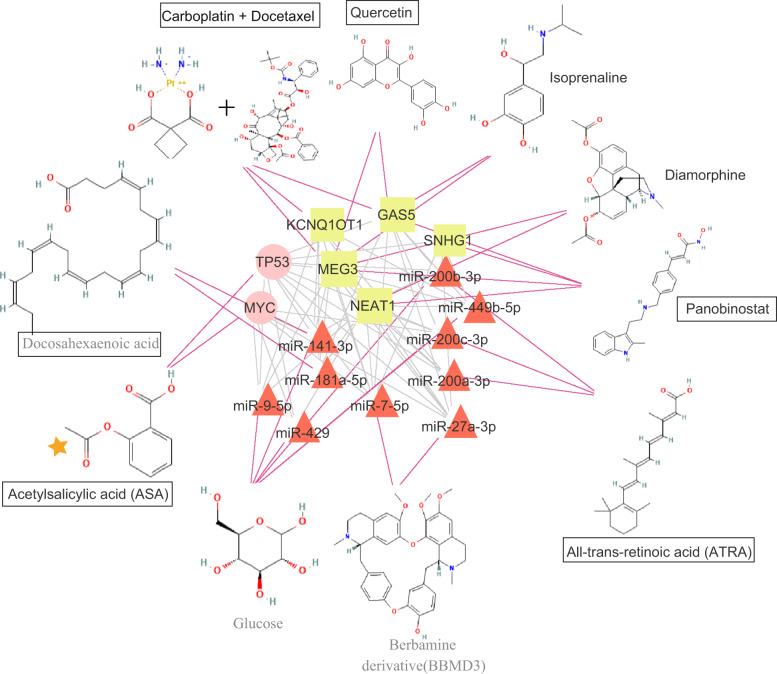
Drug repositioning prediction of high-confidence susceptibility genes.

To investigate whether these novel UM-related SGs could serve as targets of existing repositioned drugs for UM therapy, enrichment analysis was performed to examine the overlap between novel UM-related SGs and targets for a specific drug. A total of 17 novel UM-related SGs were identified as potential targets of 10 drugs, including 7 Food and Drug Administration-approved drugs. As shown in Fig. 6, acetylsalicylic acid (ASA) was predicted to target *MYC* and *TP53*. Four drugs [docosahexaenoic acid (DHA), glucose, berberine derivative and all-trans-retinoic acid (ATRA)] were predicted to target 10 novel UM-related risk miRNAs (*miR-141-3p, miR-181a-5p, miR-9-5p, miR-429, miR-200b-3p, miR-449b-5p, miR-200c-3p, miR-200a-3p, miR-7-5p*, and *miR-27a-3p*). Five drugs (carboplatin + docetaxel, quercetin, isoprenaline, diamorphine, and panobinostat) were predicted to target five novel UM-related risk lncRNAs (growth arrest-specific 5, small nucleolar RNA host gene 1, KCNQ1 opposite strand/antisense transcript 1, maternally expressed 3, and nuclear paraspeckle assembly transcript 1). Moreover, we found that four drugs (carboplatin + docetaxel, quercetin, isoprenaline, and panobinostat) targeted the same two lncRNAs (*GAS5* and *MEG3*), and two drugs (glucose and ATRA) targeted the same two miRNAs (*miR-200b-3p* and *miR-200c-3p*), in which the first two level of Anatomical Therapeutic Chemical (ATC) codes of carboplatin, docetaxel, quercetin, and ATRA were L01 (antineoplastic agents). These results indicated that if one drug targeted more predicted UM-related SGs, it was more likely to be an antineoplastic agent.

## Discussion

The biology of UM is extremely complicated, and there are currently no definitive treatments for metastatic UM. Considering the current precision medicine approaches to early diagnosis and treatment, better strategies are required to discover new SGs and candidate drugs.

Few SGs have been experimentally verified, due to the generally laborious and expensive traditional biological experiments required. Previous studies have revealed that the cytogenetic alterations on chromosomes 1p, 3, 6, and 8 are indicators of UM prognosis and metastasis^11,12^. The two mutually exclusive activating mutations in G protein alpha subunits (*GNAQ* and *GNA11*) play a crucial role, as they could interfere with the downstream signaling of G-protein-coupled receptors through the MAPK, PI3K/Akt, and Rho GTPase pathways in UM cells^7,13^. However, the clinical trials of targeted therapies based on these pathways failed to show their effects on suppressing tumor growth or improving PFS^7,8^. Therefore, pathological genomics should be explored further based on the previous findings from preclinical studies and using an efficient network to obtain a deeper understanding of the gene-protein interactions in metastatic cancer.

With advances in high-throughput sequencing and multi-omics studies, the complex relationships among multiple omics layers have been elucidated and present new opportunities for studying complex diseases^14^. Systematic network biology approaches, such as random walk and diffusion processes, have been proposed and widely applied across multiple biological layers to facilitate the discovery of genes involved in diseases;^15–17^ however, in the case of UM, they are still in their infancy. In the present study, a computational framework via propagation modeling on integrated multi-layered molecular networks was proposed to identify potential SGs and drugs for UM diagnosis, prognosis, and treatment. Under the LOOCV experiments, the iUMRG achieved superior predictive performance and yielded a higher area under the AUC value (0.8825) for experimentally verified SGs. In addition, using experimentally verified SGs as seeds, genome-wide screening was performed to detect novel candidate HSGs using the iUMRG. Multi-perspective validation analysis indicated that most of the top 50 HSGs were markedly associated with UM carcinogenesis, progression and outcome. Several candidate SGs have been repeatedly shown to be relevant to UM in multicenter cohorts. For example, *SNAI1* is a key regulator of epithelial-mesenchymal transition (EMT)^18^, and has recently been reported to be expressed in uveal melanoma lines^19^. *RelA* is a critical component of NFκB pathways active in both primary and metastatic UM^20^. *EZH2* is a known target for cancer treatment and the functional roles in many cancer types have been revealed^21^. Still, until recently, *EZH2* was found to also have tumorigenic properties in UM^22^.

Drug repositioning prediction of novel UM-related SGs yielded 17 targets and 10 drugs. Six drugs (ASA, DHA, ATRA, carboplatin + docetaxel, panobinostat and quercetin) have been approved for UM treatment. A recent study found that ASA significantly inhibited UM cell proliferation, invasion, and migration, demonstrating the potential of ASA as an adjuvant therapy drug for metastatic UM^23^. ATRA is a non-conventional anti-tumor agent that has recently been used in the treatment of UM^24^. A recent phase II clinical trial showed the association of docetaxel combined with carboplatin and overall efficacy for patients with UM^25^. In a study by Faiao-Flores et al. the pan-histone deacetylase inhibitor panobinostat was identified as a promising strategy for limiting MEK inhibitor resistance in advanced UM^26^.

This study had certain limitations that should be acknowledged. First, integrated multi-layered molecular networks may be more complete if they use varied information, such as genome-wide association data, genome-scale chromosome conformation capture (Hi-C) data, and disease similarity data. Secondly, a suitable propagation model may achieve superior predictive performance. Finally, the HSGs predicted by the iUMRG need to be further experimentally validated in vivo or in vitro.

In conclusions, the present study built an HMMN by integrating multiple types of data and proposed a computational framework via propagation modeling, which has been proven to predict novel HSGs and potential drugs for the personalized diagnosis, prognosis and treatment of UM. The iUMRG proposed in this study may serve as an effective supplementary tool in UM precision medicine and may assist the development of new medical therapies and the discovery of new SGs.

## Methods

### HMMN data

Known lncRNA-mRNA interaction relationships were obtained from LncRNATarget^27^ and starBase^28^. Experimentally supported microRNA (miRNA/miR)-target (mRNAs and lncRNAs) interactions were obtained from miRTarBase^29^ and lncBase^30^. Known transcription factor (TF)-target gene regulatory associations were obtained from TRANSFAC and TranmiR^31^. A reference map of the human binary protein interactome was downloaded from The Human Reference Interactome (HuRI)^32^. Known cancer gene sets were obtained from COSMIC^33^ and MsigDB^34^. All these data resources were summarized in Supplementary Table 1.

### UM molecular and patient datasets

59 experimentally supported UM-related SGs, including mRNAs, miRNAs and lncRNAs, were retrieved from the Comparative Toxicogenomics Database^35^, the Human microRNA Disease Database^36^, miRCancer^37^, lnc2Cancer^38^, LncRNADisease^39^, and Nc2Eye^40^.

Transcriptomics and clinical data were obtained from The Cancer Genome Atlas (TCGA) GDC Data Portal for 80 patients with UM, and Gene Expression Omnibus (GEO) database for 59 patients from the GSE44295 dataset^41^, 63 patients from the GSE22138 dataset^42^ and 28 patients from the GSE84976 dataset^43^.

These data resources were summarized in Supplementary Table 1 and Supplementary Table 2.

### Computational framework for detecting SGs via propagation modeling on integrated multi-layered molecular networks

The workflow of a computational framework (hereinafter referred to as iUMRG) via propagation modeling for detecting SGs in UM is illustrated in Fig. 1. First, an HMMN was re-constructed by integrating known lncRNA-mRNA, miRNA-mRNA, miRNA-lncRNA, TF-target and protein-protein networks. Secondly, 59 known UM-related SGs were mapped into the HMMN as seed nodes. Thirdly, a random walk with restart-based network propagation model was constructed on the HMMN to infer novel SGs as follows:

$$RG_t = r \times W^{m \times m} \times RG_{t - 1} + (1 - r) \times RG_0$$

where *W*^*m×m*^ is the adjacency matrix of the HMMN, *m* is the number of nodes in the HMMN, *RG*_0_ is the initialization vector with length *m*, in which the values of the known UM-related SGs (seed nodes) are 1/*n* (*n* is the number of seed nodes, where *n* is 59) and the values of the other nodes are 0, *r* is the restart probability (here *r* is 0.4), and *RG*_*t*_ is the equilibrium probability of each node after *t* iterations. When the procedure has reached a steady-state, *RG*_*t*_ represents the similarity of each node to the seed nodes.

The leave-one-out cross-validation (LOOCV) experiment was performed to evaluate the performance of the iUMRG. For each LOOCV trial, 58 UM-related SGs were used as seed nodes and the remaining one UM-related SG was considered as the testing case. The iUMRG was then used to calculate a risk score for each candidate node (excluding 58 seed nodes) in the HMMN. All candidate nodes were ranked according to their risk scores, which generated a ranking list for all candidate nodes. This procedure was repeated 59 times. All generated ranking lists derived from LOOCV were combined to evaluate the performance of the iUMRG. For a specified risk threshold, the true-positive rate was the fraction of correctly predicted known SGs, and the false-positive rate was the fraction of predicted unknown genes. Finally, a receiver operating characteristic (ROC) analysis was performed by varying the rank threshold to examine the performance of the iUMRG in predicting SGs.

### Drug repositioning prediction

Human drug target data was collected from drugBank^44^. Experimentally validated small molecule-miRNA and drug-lncRNA associations were retrieved from SM2miR^45^ and D-lnc^46^. All these data resources were summarized in Supplementary Table 1. The hypergeometric test was used to predict potential drugs to test the overlap between targets of specific drugs and candidate SGs. Drugs that significantly targeted candidate SGs were considered as potentially promising candidates for UM treatment.

### Statistical analyses

Differential expression analysis for genes was conducted using the R package ‘limma’. Multivariate Cox regression analysis was used to evaluate the association between SGs and OS. Kaplan–Meier survival curves were used to estimate OS and a log-rank test was used to assess the statistical significance of differences in OS between different patient groups. The optimal survival risk cutoff threshold was determined using the function ‘surv_cutpoint’ in the R package ‘survminer’. Kyoto Encyclopedia of Genes and Genomes (KEGG) functional enrichment analysis was performed and visualized using Metascape^47^.

## Supplementary information

Supplementary Table 1

Supplementary Table 2

Supplementary Table 3

## Data Availability

No datasets were generated during the current study. Data resources used in this study were summarized in Supplementary Tables 1 and 2.

## Code availability

The code used to generate the findings in this study have been deposited on GitHub (https://github.com/ZhouSunLab-Workshops/iUMRG.git).
